# Barley Brassinosteroid Mutants Provide an Insight into Phytohormonal Homeostasis in Plant Reaction to Drought Stress

**DOI:** 10.3389/fpls.2016.01824

**Published:** 2016-12-02

**Authors:** Damian Gruszka, Anna Janeczko, Michal Dziurka, Ewa Pociecha, Jana Oklestkova, Iwona Szarejko

**Affiliations:** ^1^Department of Genetics, Faculty of Biology and Environment Protection, University of SilesiaKatowice, Poland; ^2^Franciszek Gorski Institute of Plant Physiology, Polish Academy of SciencesKrakow, Poland; ^3^Department of Plant Physiology, University of Agriculture in KrakowKrakow, Poland; ^4^Laboratory of Growth Regulators, Faculty of Science, Centre of the Region Haná for Biotechnological and Agricultural Research, Institute of Experimental Botany, Czech Academy of Sciences, Palacký UniversityOlomouc, Czechia

**Keywords:** barley, brassinosteroids, drought, homeostasis, mutants, phytohormones

## Abstract

Brassinosteroids (BRs) are a class of steroid phytohormones, which regulate various processes of morphogenesis and physiology—from seed development to regulation of flowering and senescence. An accumulating body of evidence indicates that BRs take part in regulation of physiological reactions to various stress conditions, including drought. Many of the physiological functions of BRs are regulated by a complicated, and not fully elucidated network of interactions with metabolic pathways of other phytohormones. Therefore, the aim of this study was to characterize phytohormonal homeostasis in barley (*Hordeum vulgare*) in reaction to drought and validate role of BRs in regulation of this process. Material of this study included the barley cultivar “Bowman” and five Near-Isogenic Lines (NILs) representing characterized semi-dwarf mutants of several genes encoding enzymes participating in BR biosynthesis and signaling. Analysis of endogenous BRs concentrations in these NILs confirmed that their phenotypes result from abnormalities in BR metabolism. In general, concentrations of 18 compounds, representing various classes of phytohormones, including brassinosteroids, auxins, cytokinins, gibberellins, abscisic acid, salicylic acid and jasmonic acid were analyzed under control and drought conditions in the “Bowman” cultivar and the BR-deficient NILs. Drought induced a significant increase in accumulation of the biologically active form of BRs—castasterone in all analyzed genotypes. Another biologically active form of BRs—24-epi-brassinolide—was identified in one, BR-insensitive NIL under normal condition, but its accumulation was drought-induced in all analyzed genotypes. Analysis of concentration profiles of several compounds representing gibberellins allowed an insight into the BR-dependent regulation of gibberellin biosynthesis. The concentration of the gibberellic acid GA_7_ was significantly lower in all NILs when compared with the “Bowman” cultivar, indicating that GA_7_ biosynthesis represents an enzymatic step at which the stimulating effect of BRs on gibberellin biosynthesis occurs. Moreover, the accumulation of GA_7_ is significantly induced by drought in all the genotypes. Biosynthesis of jasmonic acid is also a BR-dependent process, as all the NILs accumulated much lower concentrations of this hormone when compared with the “Bowman” cultivar under normal condition, however the accumulation of jasmonic acid, abscisic acid and salicylic acid were significantly stimulated by drought.

## Introduction

Brassinosteroids (BRs) are a class of polyhydroxylated steroid phytohormones, which regulate various processes of plant morphogenesis and physiology—from seed development and germination up to regulation of flowering and senescence (Choudhary et al., 2012; Vriet et al., 2012). An accumulating body of evidence indicates that the regulation of such vast aspects of plant physiology is mediated through a network of molecular interactions between BR metabolism and signalosomes of other phytohormones (Nemhauser et al., 2006; Hansen et al., 2009; Domagalska et al., 2010; Chung et al., 2011; Gruszka, 2013). Studies on BR metabolism, which have been conducted for the last 30 years mainly in the model plant species *Arabidopsis thaliana*, led to identification of numerous components of BR biosynthesis and signaling pathways (Vriet et al., 2012), however the regulation and the course of these processes are far less understood in crop species, including barley.

Research on molecular mechanisms of BR metabolism in barley led to identification of a few genes only. One of the genes with validated function is *HvBRI1*, encoding a BR receptor (Chono et al., 2003; Saisho et al., 2004; Gruszka et al., 2011a), whereas only few genes: *HvDWARF, HvBRD, HvCPD*, and *HvDIM* encoding enzymes mediating BR biosynthesis were identified and functionally analyzed (Gruszka et al., 2011b; Dockter et al., 2014). However, it should be noted that many aspects of BR biosynthesis and its regulation in barley are still poorly understood (Gruszka et al., 2016). It was reported that some of the newly identified semi-dwarf barley mutants showing defects in BR signaling or biosynthesis may serve as an alternative in breeding programs, especially in face of the global climate change (Dockter et al., 2014). However, research on physiological reaction of the barley semi-dwarf BR mutants to drought stress has been conducted only recently. Mutants deficient in BR metabolism are very valuable tools to study the mechanism of action of these hormones also in response to stress conditions (Janeczko et al., 2016).

Apart from their role in regulation of plant growth and development, BRs have also been implicated in a control of plant stress responses (Nakashita et al., 2003; Xia et al., 2009; Deb et al., 2016). Abiotic stresses pose a serious threat to crop yields. Drought belongs to the major stresses in agriculture (Mahajan and Tuteja, 2005) resulting in serious loss in crop production and shortage of food supplies (Wang et al., 2003; Ferrero-Serrano and Assmann, 2016). Moreover, it is suggested that in several regions of the world crop losses caused by drought will become even more evident (Llanes et al., 2016). During evolution plants have developed a broad range of adaptive reactions, including morphological, physiological and molecular mechanisms allowing a proper response to drought stress (Ahmadi et al., 2010). One of the most important mechanisms is alteration in endogenous phytohormone concentrations (Llanes et al., 2016). However, in previous studies the influence on plant physiological reactions to drought was analyzed mainly through exogenous BR treatment or application of BR biosynthesis inhibitor, consequently there is still a lack of direct genetic evidence for a role of endogenous BRs in modulation of plant stress responses (Jager et al., 2008; Divi et al., 2010; Zhou et al., 2014). It should be also kept in mind that endogenous BRs do not seem to undergo a long-distance transport in plants, and that exogenous BRs are not transported upon application by spraying (Symons et al., 2008; Janeczko and Swaczynova, 2010). Moreover, recent reports indicate that reduction of BR responses improves plant tolerance to drought (Northey et al., 2016). This finding is somewhat incongruent with the previous reports indicating that exogenous BR application increases plant tolerance to abiotic stresses (Kagale et al., 2007; Bajguz and Hayat, 2009; Yuan et al., 2010). However, it should be kept in mind that during these studies exogenous BR was applied at high concentrations, which are known to induce various physiological responses (Northey et al., 2016).

Genetic analyses indicated an antagonistic role for endogenous BR in abscisic acid (ABA) responses, which are crucial for the stress tolerance (Rodrigues et al., 2009; Krasensky and Jonak, 2012; Li et al., 2012; Chung et al., 2014; Ryu et al., 2014). ABA inhibits action of growth-promoting hormones, including BR signaling, and masks BR effects in plant stress responses (Bajguz and Hayat, 2009; Divi et al., 2010). It has also been reported that BR mutants are more sensitive to ABA treatment (Choe et al., 2002). It was suggested that the stress tolerance mechanisms limit energy-requiring BR-dependent growth processes. However, the regulatory mechanisms, which control ABA and BR activity remain largely unknown, particularly in water-stressed plants (Chung et al., 2014; Janeczko et al., 2016). It has been postulated that BR and ABA responses remain in a constant balance, regulating homeostasis and plant development. Application of excessive exogenous BR may stimulate ABA accumulation, thereby preventing enhanced BR responses within some feedback loop. The other way round, a deficiency in BR biosynthesis may result in hypersensitization to endogenous ABA, and consequently to an enhanced drought tolerance (Northey et al., 2016). It is known that BR signaling antagonistically regulates stress response genes (Kim et al., 2010). It was also reported that BR-overproducing and constitutive BR signaling mutants are less tolerant to stresses, what suggests that BR negatively regulates stress responses (Chung et al., 2012, 2014; Kim et al., 2013). On the other hand, stress responsive transcription factors appear to limit metabolic energy dedicated to growth in order to enhance stress tolerance through downregulating BR responsive genes (Chung et al., 2014).

There are several phytohormones including ethylene, jasmonic acid (JA), salicylic acid (SA), and ABA with validated role in plant adaptation to biotic and abiotic stresses, among which ABA is a key plant hormone regulating physiological reaction to drought (Zhu, 2002; Bari and Jones, 2009; Krasensky and Jonak, 2012). It is known that many of the physiological functions regulated by BRs are in fact modulated based on a complicated network of interactions with other hormones: auxin (Hardtke et al., 2007; Jung et al., 2010), gibberellin (Shimada et al., 2006; Bai et al., 2012; Gallego-Bartolomé et al., 2012; Unterholzner et al., 2015), ABA (Steber and McCourt, 2001; Zhang et al., 2009), ethylene (Yi et al., 1999), and jasmonic acid (Ren et al., 2009; Gan et al., 2015). This interaction is based on some crosstalk between BR biosynthesis and signal transduction pathways and signalosomes of other phytohormones (Gruszka, 2013; Saini et al., 2015). The inter-hormonal crosstalk constitutes an additional level of complexity in physiological responses to various stresses, and provides a mechanism to control system robustness and dynamicity (Deb et al., 2016). However, this relationship has been documented mainly with respect to plant growth regulation, and there are very few reports describing the interaction between BR and other phytohormones in the control of stress response mechanisms (Divi et al., 2010; Chung et al., 2014). Most of the stress hormones-responsive genes are also regulated by BR, which constitutes another area of inter-hormonal crosstalk. Moreover, BR may promote some anti-stress mechanisms, that are independent of ABA, ethylene, jasmonic acid and salicylic acid at least to some extent (Divi et al., 2010). It additionally complicates the whole mechanism, and requires further research. Taking this aspect into account, it seems important to analyze and characterize physiological reaction of the BR mutants to drought. Since many physiological processes, including the stress response, are regulated by coordinated action of various phytohormones, the present study was performed by analysis of hormonal homeostasis in these mutants and in a reference variety under control and drought conditions, in order to get an insight into the role of BRs in plant responses to drought, and regulation of the hormonal homeostasis.

## Materials and methods

### Plant material

The plant material of this study included the barley (*Hordeum vulgare*) two-rowed spring-type cultivar “Bowman” and a group of semi-dwarf Near-Isogenic Lines (NILs), representing the previously characterized barley mutants with defects in BR biosynthesis and signaling (Dockter et al., 2014). These NILs belong to a broad collection, which was developed by recurrent crossing of original mutants into the homogeneous genetic background of the barley “Bowman.” The “Bowman” near-isogenic lines have also been genotyped with up to 3000 markers, which provided information on isogenicity and approximate map position of each introgression region (Druka et al., 2011; Salvi et al., 2014). As a result of this approach each of the NILs harbors a restricted and mapped genomic introgression region, specific and derived from a given original mutant in the homogeneous genetic background of the “Bowman” cultivar, which is shared by all the NILs. This approach enables and simplifies comparative evaluation of the mutants and physiological analysis (Salvi et al., 2014). Grain of these genotypes are either available from the Nordic Genetic Resource Center (http://www.nordgen.org) or from the National Small Grains Collection (http://www.ars.usda.gov).

Apart from the reference “Bowman” cultivar, the plant material of this study includes the following NILs showing defects in BR biosynthesis: BW084 (*brh13.p*) carrying a missense mutation in the *HvCPD* gene, BW091 (*brh3.g*) in which a nonsense mutation was identified in the *HvBRD* gene, and the line BW333 (*ert-zd.159*) harboring a missense mutation in the *HvDIM* gene. Additionally, the plant material includes the following NILs characterized by defects in BR perception: BW312 (*ert-ii.79*) and BW885 (*uzu1.a*) carrying missense mutations in different domains of the HvBRI1 BR receptor. Details on the identified mutations were published by Dockter et al. (2014). Various molecular, genetic and physiological analyses, coupled with measurement of endogenous BR concentration in these NILs indicated that their phenotype is caused by perturbations in BR biosynthesis or signaling. It should be noted, that the characterized abnormalities in BR metabolism result in phenotypic alterations only in the above-ground part of the barley mutants causing the semi-dwarf stature, however having no effect on root architecture of these mutants (Dockter et al., 2014), therefore the selected NILs constitute an ideal material for research on influence of the perturbations in BR metabolism on physiological reaction of the mutants to drought.

### Experimental design and plant material sampling

Details on preparation of soil mixture and the experiment setup as well as calculations aimed at determining the maximum (100%) soil water capacity were described by Janeczko et al. (2016). Seeds of the “Bowman” cultivar and the analyzed NILs were sown in Petri dishes (diameter 10 cm, number of seeds per dish: 45) on moistened filter paper and kept in the dark (24°C). On day 4th, the germinated seeds were transferred to pots (15 cm × 15 cm × 38 cm) with the prepared soil mixture (10 seedlings per pot) and transferred to a growth chamber. The growth conditions were the following: 12 h-photoperiod, light intensity (130 μmol m^−2^ s^−1^), temperature on the days 4–7 of the experiment 23°C (day/night), temperature on the days 8–28 of the experiment 18°/15°C (day/night). All plants were watered optimally (70% of soil water capacity) until day 28th of the experiment. On the next day (29th) of the experiment, plants of each genotype (the “Bowman” cultivar and NILs) were divided into two groups and the temperature in growth chamber has been set at 22°/18°C (day/night). The first group of plants was watered optimally until the end of the experiment. The plants from the second group were grown for 15 days under conditions of an increasing water deficit due to watering cessation. In the end of drought period the soil water capacity was at the level 25% and leaf wilting was visible. Technical details of procedure of obtaining and controlling of the water deficit are given in the article by Janeczko et al. (2016). Leaf-tissue samplings for the extractions and measurements of the endogenous concentrations of the phytohormones were performed on the day 38th of vegetation (group of watered plants) and on the day 45th (drought-exposed plants). During the leaf-tissue samplings plants of all the genotypes were at the same developmental stage (fifth leaf). The fully developed leaves (third and fourth) were collected for the analysis. For the measurement of the endogenous concentrations of BRs 1 g of fresh weight samples were prepared, where one sample contained two leaves from two different plants. For the measurement of the endogenous contents of the other phytohormones 2 g of fresh weight samples were prepared, where one sample contained four leaves from four different plants.

### Extraction and quantification of endogenous brassinosteroids

Extraction of endogenous BRs was preceded by homogenization of leaf tissue (1 g F.W.) in 20 ml 80% methanol (MeOH) for 12 h at 4°C and the homogenates were then centrifuged (36 670 g, 10 min, 4°C). The supernatants were supplemented with internal standard mixture containing 10 pmol each of ^2^H_3_-labeled BRs and then loaded on the Discovery® DPA-6S columns (Supelco, Bellefonte, PA 16823, USA) (Tarkowská et al., 2016). The filtered volumes were evaporated to dryness *in vacuo*. Each sample residue was then dissolved in 75 μL of 100% MeOH by vortexing and sonicating for 5 min, and made up to 1 ml with PBS buffer (50 mM NaH_2_PO_4_ and 15 mM NaCl, pH 7.2). The samples were loaded on columns containing antibodies against BRs (IAC columns). BRs from the IAC columns were eluted using 3 mL of ice-cold MeOH (−20°C). The elution fraction was evaporated to dryness in a benchtop concentrator (CentriVap® Acid-Resistant benchtop concentrator, Labconco Corp., MO, USA) and analyzed by Ultra High Performance Liquid Chromatography UHPLC-MS/MS (Tarkowská et al., 2016). Each sample represents leaf tissue (3rd and 4th leaf) collected from two individual plants of a given genotype. Each of the analyses was performed in triplicates.

### Extraction and quantification of endogenous abscisic acid (ABA), auxins, cytokinins (CKs), gibberellins (GAs), jasmonic acid (JA), and salicylic acid (SA)

Extraction and quantifications of the plant hormones (ABA, auxin, CKs, GAs, JA, and SA) were performed as described in the article by Dziurka et al. (2016). Briefly, lyophilized leaf samples after pulverization were transferred to methanol:water:formic acid mixture (15:4:1 v/v). To each sample internal isotopic standards of the phytohormones (OlChemIm, Olomouc, Czech Republic) were applied. After centrifugation, the supernatant was evaporated to dryness, and the residue was suspended in 1 M aqueous solution of formic acid. Samples were passed through the activated SPE cartridges (BondElute Plexa PCX, Agilent, USA). The fraction of neutral and acidic hormones (ABA, auxins, GAs, JA, SA) was eluted with methanol:acetonitrile (1:1 v/v), the basic compounds (CKs) were eluted with 5% ammonia in methanol:acetonitrile (1:1 v/v). All samples were evaporated to dryness, resuspended in 100 μl of methanol, filtered and used for UHPLC analyses. The endogenous concentrations of hormones were measured on the UHPLC apparatus (Agilent Infinity 1260, Agilent, Germany) coupled to a triple quadruple mass spectrometer (6410 Triple Quad LC/MS, Agilent, USA) with electrospray ionization (ESI). The exact conditions of the analysis are given in the article by Dziurka et al. (2016). Standards of the phytohormones were purchased from OlChemIm (Olomouc, Czech Republic). Solvents of the HPLC grade were purchased from Sigma-Aldrich (Poznan, Poland). Each of the analyses was performed three times (samples from the optimally watered plants) and four times (samples of the drought-stressed plants).

### Analysis of plant response to drought

Leaf gas exchange was measured using the Li-6400 infrared gas analyzer (Licor Inc., Lincoln, USA) equipped with a standard leaf chamber and a LED light source. The CO_2_ concentration during the measurement was 380 ppm. The measurements were performed before the leaf-tissue samplings, on the day 38th of vegetation (group of watered plants) and on the day 45th (drought-exposed plants). The following parameters were recorded: net photosynthesis rate (Pn), transpiration rate (E), and stomatal conductance (gs). The measurements were carried out on well-developed leaves in seven replicates/genotype, where one replicate represented one leaf from an individual plant. Index of greenness of well-developed leaves, informing about chlorophyll content, was measured using the Minolta SPAD Chlorophyll Meter 502P (Spectrum Technologies, Inc., USA). The measurements were performed in 10 replicates/genotype (one replicate represented one leaf from an individual plant).

### Statistical analysis

Representation of individual plants of a genotype within each sample and the number of replicates for each analysis/measurement is given in the method descriptions above. Statistical significance was calculated using Duncan's test (*P* ≤ 0.05). Calculation was made separately for the optimally watered group of plants (control) and drought-stressed group of plants (comparison of the averages obtained in a particular analysis for the “Bowman” cultivar and the analyzed NILs within the control and drought-stressed group).

## Results

### Profile of brassinosteroid accumulation and its alteration in response to drought

**Figure 1:**
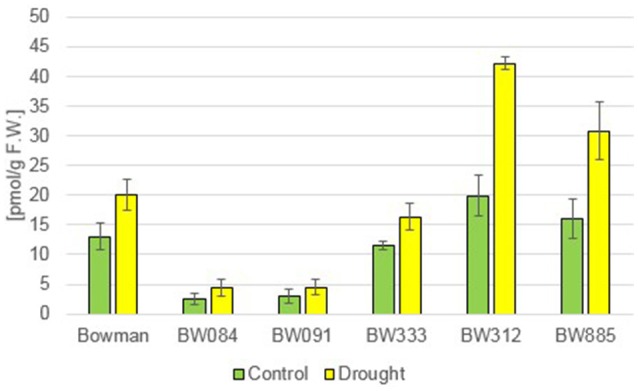
**The endogenous concentrations of castasterone (CS) in the analyzed genotypes under the control and drought conditions**. The mean values of three replicates of each measurement are presented for each genotype, with error bars representing standard deviation.

The measurements of the endogenous BRs concentration in the “Bowman” cultivar and the analyzed NILs under the control condition (optimal watering) confirmed that the phenotype of the NILs is caused by the mutations identified by us previously: as far as castasterone (CS) is concerned, the BR-deficient genotypes showed decreased concentrations of this compound, whereas BR-insensitive NILs showed a significantly raised content of this compound with respect to the “Bowman” cultivar (Figure 1). Interestingly, in this research we confirmed that the endogenous CS accumulation is significantly higher in the BR-insensitive BW312 (*ert-ii.79*) line than in the allelic line BW885 (*uzu1.a*). The BW312 line harbors the Thr-573 to Lys amino acid substitution within the BR ligand-binding island domain of the HvBRI1 receptor. The BW885 line carries the His-857 to Arg substitution within the kinase domain of the BR receptor. Moreover, this difference in the accumulation of CS in these two BR-insensitive lines was observed under the control and drought conditions. It should be pointed out that in reaction to drought the endogenous CS accumulation increased significantly in all analyzed genotypes, however the drought-stimulated increase in the CS content was most prominent in the BR-insensitive lines BW312 and BW885 (Figure 1). This indicates that CS, as the suggested end product of the BR biosynthetic pathway in monocots, may be referred to as the stress-induced compound. Moreover, this drought-induced physiological adaptation is retained in both the BR-deficient and BR-sensitive mutants, in which the drought-induced increase in CS accumulation also occurred. In the present study 24-epicastasterone (24-epiCS) has not been detected in any of the analyzed genotypes, irrespective of the growth condition.

**Figure 2:**
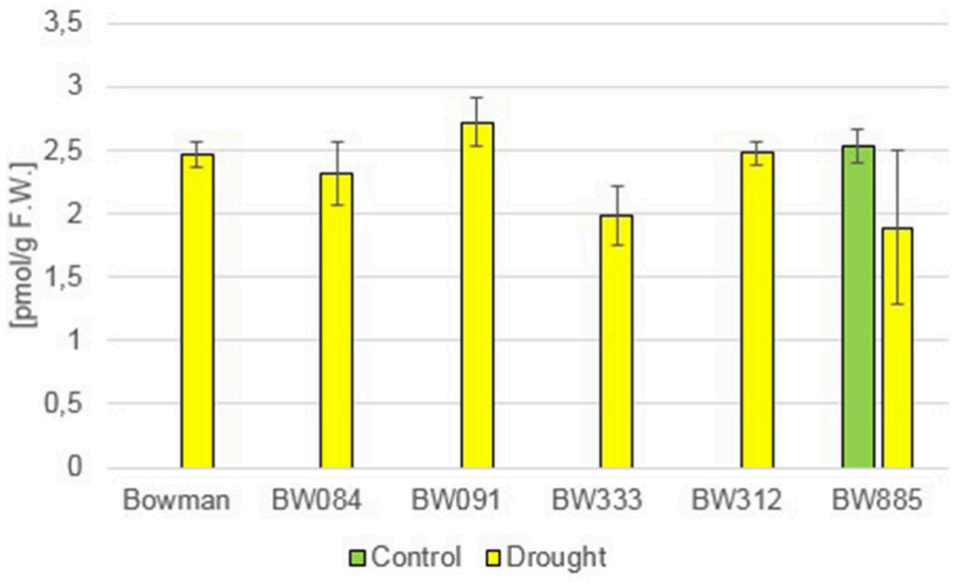
**The endogenous contents of 24-epibrassinolide (24-epiBL) in the analyzed genotypes under the control and drought conditions**. The mean values of three replicates of each measurement are presented for each genotype, with error bars representing standard deviation.

Brassinolide (BL) has also not been detected in any of the analyzed genotypes. However, a relatively low accumulation of another compound—24-epibrassinolide (24-epiBL)—has been detected specifically in the BR-insensitive line BW885 under control conditions (Figure 2). Moreover, accumulation of this compound was apparently stimulated in all the genotypes as a results of the drought stress (Figure 2). This suggests that 24-epiBL is, apart from CS, another representative of BRs, whose accumulation is stimulated by drought. Interestingly, the accumulation of 24-epiBL was drought-induced to roughly the same extent in all the genotypes (the “Bowman” cultivar and all analyzed NILs), and with no significant difference between the BR-deficient and BR-insensitive genotypes. It indicates that proficiency in BR biosynthesis or signaling is not required for the drought-induced increase in the 24-epiBL accumulation.

**Figure 3:**
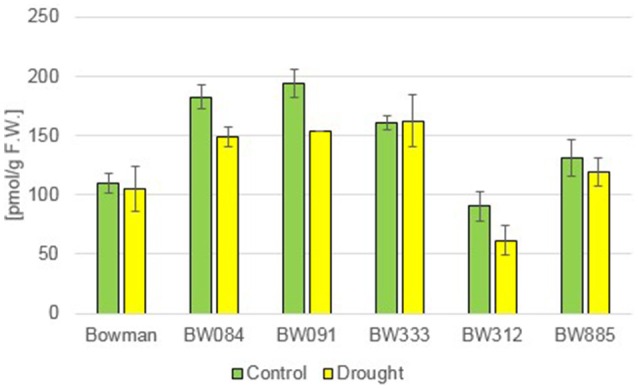
**The endogenous concentrations of 28-homocastasterone (28-homoCS) in the analyzed genotypes under the control and drought conditions**. The mean values of three replicates of each measurement are presented for each genotype, with error bars representing standard deviation.

Additionally to the C_28_ BRs described above, representative of the C_29_ BRs, 28-homocastasterone (28-homoCS), has been detected in relatively large quantities in all analyzed genotypes. The accumulation of this compound was observed under the control and drought condition (Figure 3). However, a quite interesting phenomenon was observed when the 28-homoCS accumulation was compared between the analyzed genotypes: under the control condition a significantly higher accumulation of 28-homoCS was reported in the BR biosynthesis-deficient lines, especially in BW084 and BW091 when compared with the “Bowman” cultivar (ca. 167 and 177% of the “Bowman” reference value, respectively). In the third of the BR biosynthesis-deficient lines, BW333, the accumulation of 28-homoCS was elevated to a lesser degree (ca. 147% of the “Bowman” reference value). Another interesting observation is that the lowest concentration of 28-homoCS was observed in the BR-insensitive line BW312, in which the highest concentration of CS was observed (see above). Moreover, comparison with the other BR-insensitive line BW885, in which concentration of 28-homoCS is significantly higher, indicates that under the optimal watering condition the accumulation of 28-homoCS is inversely correlated with the accumulation of CS, and this phenomenon seems to be true for all analyzed genotypes: mutants deficient in the biosynthesis of CS accumulate the highest concentrations of 28-homoCS, on the other hand the BR-insensitive line BW312, in which the highest concentration of CS was observed, accumulates the lowest concentration of 28-homoCS. Interestingly, the drought condition did not cause any increase in the concentration of 28-homoCS in the analyzed genotypes. Both in the “Bowman” cultivar and the analyzed NILs the contents of 28-homoCS were comparable with the respective values reported under the control condition (Figure 3). This indicates that in contrast to the accumulations of CS and 24-epiBL (representing C_28_ BRs), which were significantly stimulated by drought in all analyzed genotypes, the accumulation of 28-homoCS (representing C_29_ BRs) is not induced by the stress condition.

### Drought-induced changes in accumulation of gibberellins

**Figure 4:**
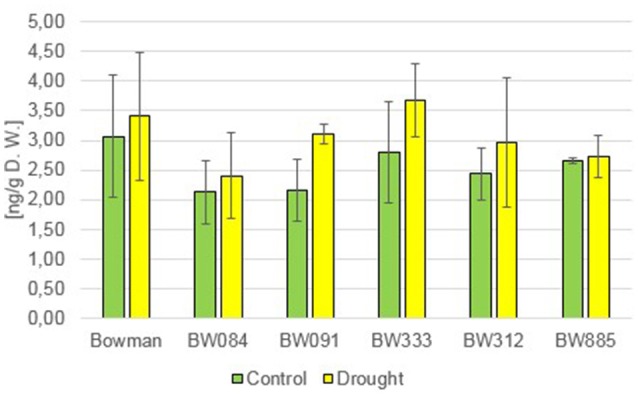
**The endogenous contents of the gibberellin GA_**4**_ in the analyzed genotypes under the control and drought conditions**. The mean values of three replicates of each measurement are presented for each genotype, with error bars representing standard deviation.

In order to get an insight into the role of BRs in regulation of the homeostasis of gibberellic acid (GA), the concentrations of several GAs were determined. Analysis of the endogenous content of GA_4_, as one of the major bioactive forms of GAs, indicated that under the control condition all analyzed NILs contained lower concentrations of this compound when compared with the “Bowman” cultivar, however these differences were not found to be statistically significant (Figure 4). The drought stress induced a slight increase in the content of GA_4_ in all analyzed genotypes (with respect to the control condition), however differences between the “Bowman” cultivar and the analyzed NILs were not statistically significant (Figure 4). The ability to react to drought by the slight increase in concentration of GA_4_ is retained in both the BR-deficient and BR-insensitive NILs, however this increase does not seem to be a major reaction of GA metabolism to drought.

**Figure 5:**
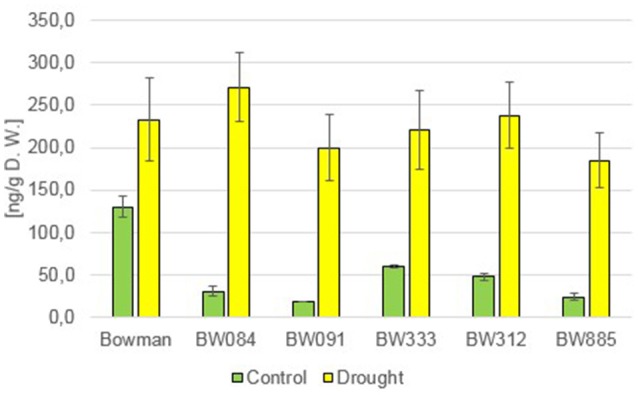
**The endogenous concentrations of the gibberellin GA_**7**_ in the analyzed genotypes under the control and drought conditions**. The mean values of three replicates of each measurement are presented for each genotype, with error bars representing standard deviation.

However, measurement of the endogenous content of another major, biologically active representative of GAs, GA_7_, led to quite interesting results. Under the control condition, all analyzed NILs showed significantly lower concentrations of this compound when compared with the “Bowman” cultivar (14.5–46% of the “Bowman” value) (Figure 5). Under the drought stress a significant increase in the endogenous concentration of GA_7_ was reported in all analyzed genotypes, however in the analyzed NILs this increase was even more apparent (371–1058% of the control values) than in the “Bowman” cultivar (179% of the control value). Consequently, under the drought conditions the contents of endogenous GA_7_ in the “Bowman” cultivar and the analyzed NILs were comparable, and the differences were statistically insignificant (Figure 5). This indicates that in barley GA_7_ is a major representative of GAs, whose concentration is drought-induced. This induction of GA_7_ accumulation is particularly significant in the BR-deficient and BR-insensitive NILs, even though under the control condition these mutants contain much lower contents of the endogenous GA_7_ when compared with the reference cultivar “Bowman.”

### In barley perturbations in BR metabolism do not affect auxin accumulation

**Figure 6:**
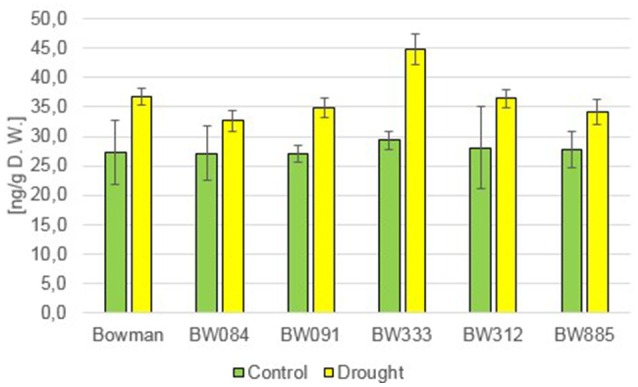
**The endogenous contents of Indole-3-Acetic Acid (IAA) in the analyzed genotypes under the control and drought conditions**. The mean values of three replicates of each measurement are presented for each genotype, with error bars representing standard deviation.

Under the control conditions, the endogenous concentration of Indole-3-Acetic Acid (IAA) was very similar in all analyzed genotypes, without any statistically significant difference (Figure 6). The drought stress induced an increase in the endogenous IAA concentrations in all analyzed genotypes (120–158% of the control values), however differences between the “Bowman” cultivar and the analyzed NILs were not significant (Figure 6). Similar results were obtained when the endogenous concentration of another representative of auxins—ChloroIndole-3-Acetic Acid (ClIAA) was analyzed. Under the control condition the endogenous concentration of this compound was very similar in all analyzed genotypes, without any statistically significant difference. The concentration of ClIAA in all the genotypes was slightly lower than IAA. However, similarly to IAA, drought induced an increase in the endogenous content of ClIAA in all analyzed genotypes (138–184% of the control values), but differences between the genotypes were only slight and not significant (data not shown). These results indicate that drought stimulates the increase in the endogenous concentrations of both representatives of auxins in the analyzed genotypes, and that BR-deficiency or BR-insensitivity do not seem to affect the auxin homeostasis in both growth conditions.

### Drought-induced changes in accumulation of abscisic acid

**Figure 7:**
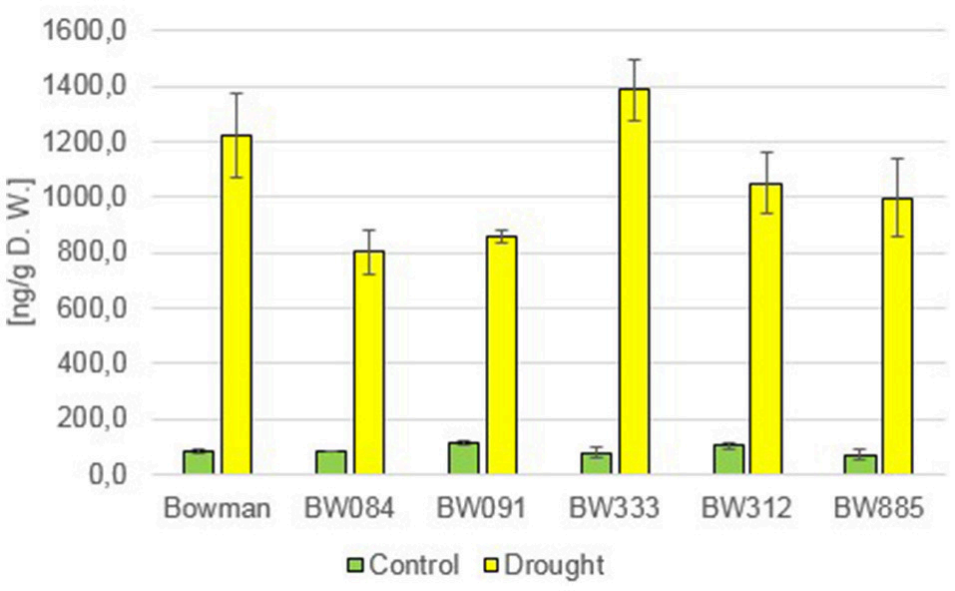
**The endogenous concentrations of abscisic acid (ABA) in the analyzed genotypes under the control and drought conditions**. The mean values of three replicates of each measurement are presented for each genotype, with error bars representing standard deviation.

Since it is known that ABA plays a pivotal role in plant responses to various abiotic stresses, including drought, in the presented research the role of BRs in the regulation of ABA homeostasis under the control and drought conditions was analyzed. When grown under optimal watering, the BR-deficient and BR-insensitive NILs accumulated similar concentrations of ABA when compared with the “Bowman” cultivar, differences reported between the analyzed NILs and the “Bowman” cultivar were not statistically significant, and no tendency was observed between the analyzed genotypes (Figure 7). As expected, the drought stress induced a very significant increase in the accumulation of ABA in all analyzed genotypes (Figure 7). The endogenous concentrations of ABA increased at least seven times in reaction to drought in all analyzed genotypes, however no tendency was observed between the BR-deficient, BR-insensitive NILs and the “Bowman” cultivar, also when a relative increase in the ABA content with regard to the control value was calculated for each genotype. These results indicate that perturbations in BR biosynthesis or BR signaling do not affect the endogenous ABA accumulation under the control condition, and do not influence the capacity of the barley BR mutants to react to drought with such a significant increase in ABA accumulation.

### Accumulation of salicylic acid under the control and drought stress conditions

**Figure 8:**
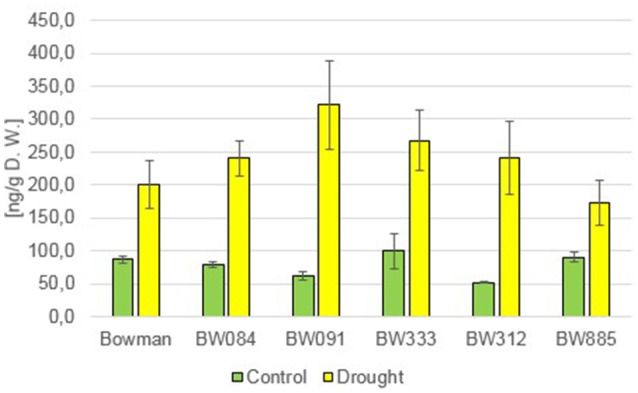
**The endogenous contents of salicylic acid (SA) in the analyzed genotypes under the control and drought conditions**. The mean values of three replicates of each measurement are presented for each genotype, with error bars representing standard deviation.

In this study here, an accumulation profile of salicylic acid (SA) as a representative of stress hormones was established. Under the control condition, most of the BR-deficient and BR-insensitive NILs contained comparable concentrations of this hormone with respect to the “Bowman” cultivar, and no tendency in differences in the SA content was observed between the analyzed genotypes (Figure 8). In reaction to drought stress, a significant increase in the endogenous content of SA was reported in all analyzed genotypes (190–514% of the control values). However, no tendency in differences in the SA content was observed between the analyzed genotypes (Figure 8). This indicates that the alteration in SA homeostasis is a physiological reaction of barley plants to drought. Moreover, the abnormalities in BR biosynthesis or signaling do not seem to affect the SA homeostasis under the control condition, and the capability of the mutants to react to the drought stress with the increase in SA accumulation.

A further confirmation that endogenous BRs do not influence the SA homeostasis resulted from a measurement of endogenous concentration of benzoic acid, which is known as a SA precursor. Under the control condition, all analyzed genotypes contained a very similar concentration of benzoic acid. Moreover, under the drought condition the endogenous concentration of benzoic acid was very similar in all analyzed genotypes as well. Interestingly, no significant alteration in the benzoic acid content was reported in any of the genotypes under the drought condition with respect to the control values (data not shown). This result allowed us to postulate that the drought-stimulated, significant increase in the endogenous content of SA occurs, at least in barley, at a step downstream of benzoic acid in the SA biosynthesis pathway.

### In barley regulation of jasmonic acid homeostasis is brassinosteroid-dependent

**Figure 9:**
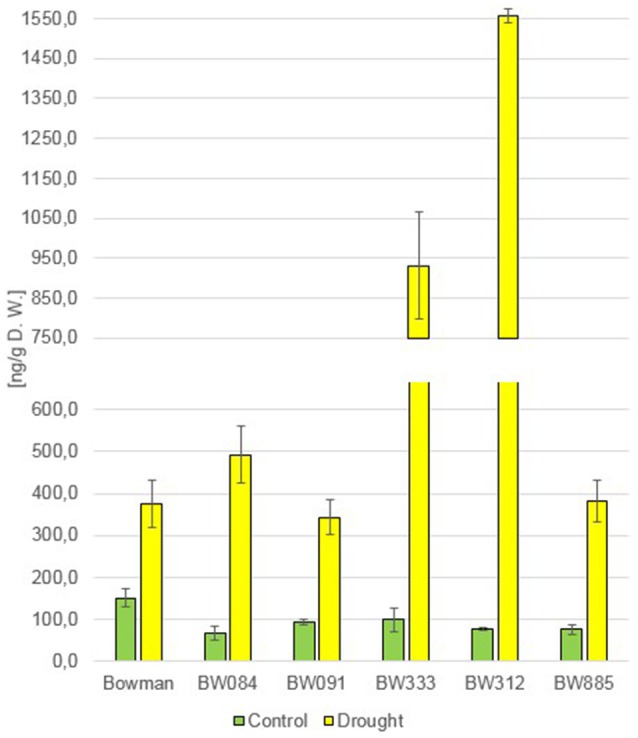
**The endogenous concentrations of jasmonic acid (JA) in the analyzed genotypes under the control and drought conditions**. The mean values of three replicates of each measurement are presented for each genotype, with error bars representing standard deviation.

The measurement of endogenous jasmonic acid (JA) accumulation under the control condition indicated that all analyzed NILs (both BR-deficient and BR-insensitive) contained significantly lower concentrations of this hormone when compared with the “Bowman” cultivar (ca. 45–65% of the “Bowman” value) (Figure 9). This indicated that the JA homeostasis in barley is dependent on BR synthesis and signaling processes. Under the drought condition a significant increase in the JA content was reported in all analyzed genotypes (Figure 9). However, some differences between the genotypes were identified in a relative increase in JA concentration with respect to the control values: in the “Bowman” cultivar the drought-induced JA accumulation raised by ca. 250% with respect to the control value for this genotype. In the BW084, BW091, and BW885 NILs the drought-induced JA accumulation was elevated by ca. 730, 470, and 500%, respectively, whereas the most significant increase with respect to the control values was reported in the BW312 and BW333 NILs (ca. 1990 and 940%, respectively). In the case of the BW312 and BW333 NILs we postulate that such a significant increase in the JA accumulation may be a gene-specific (BW333) or allele-specific (BW312) phenomenon. Noteworthy, despite the fact that under the control condition all analyzed NILs contained the significantly lower concentrations of JA when compared with the “Bowman” cultivar, upon drought stress all analyzed mutants contained comparable or significantly higher concentrations of JA with respect to the “Bowman” cultivar. It indicates that both the BR-deficient and BR-insensitive barley mutants retain the capacity of significantly increasing the endogenous JA content in reaction to drought stress.

### Accumulation of cytokinins under the control and drought stress conditions

**Figure 10:**
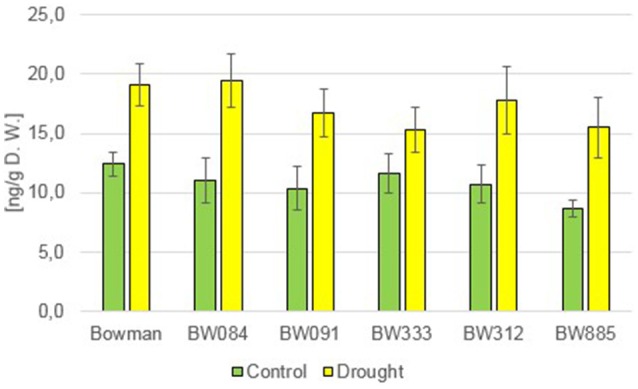
**The endogenous contents of ***trans***-Zeatin (tZ) in the analyzed genotypes under the control and drought conditions**. The mean values of three replicates of each measurement are presented for each genotype, with error bars representing standard deviation.

In order to get an insight into an accumulation profile of cytokinins (CKs), two representatives of these phytohormones were assayed: *cis*-Zeatin (*c*Z) and *trans*-Zeatin (*t*Z). As far as *c*Z is concerned, under the control condition all analyzed genotypes contained very similar endogenous concentrations of this compound. Moreover, the endogenous concentration of *c*Z was very similar among all analyzed genotypes under the drought condition as well. Interestingly, no significant alteration in the endogenous content of *c*Z was observed in any of the analyzed genotypes in reaction to drought (data not shown). The endogenous concentration of the second representative of CKs, *trans*-Zeatin, proved to be very similar in all analyzed genotypes under the control condition (Figure 10). However, contrary to *c*Z, the accumulation of *t*Z was stimulated by drought in all analyzed genotypes (130–180% of the control values). Nevertheless, under the drought condition no significant difference in the accumulation of *t*Z was reported between any of the analyzed genotypes (Figure 10). This indicates that abnormalities in BR biosynthesis or signaling in the analyzed NILs do not seem to impact the endogenous concentration of these two CKs, and do not influence the ability of the analyzed NILs to react to drought with the increase in the *t*Z accumulation.

### Characterization of plant reaction to drought

**Figure 11:**
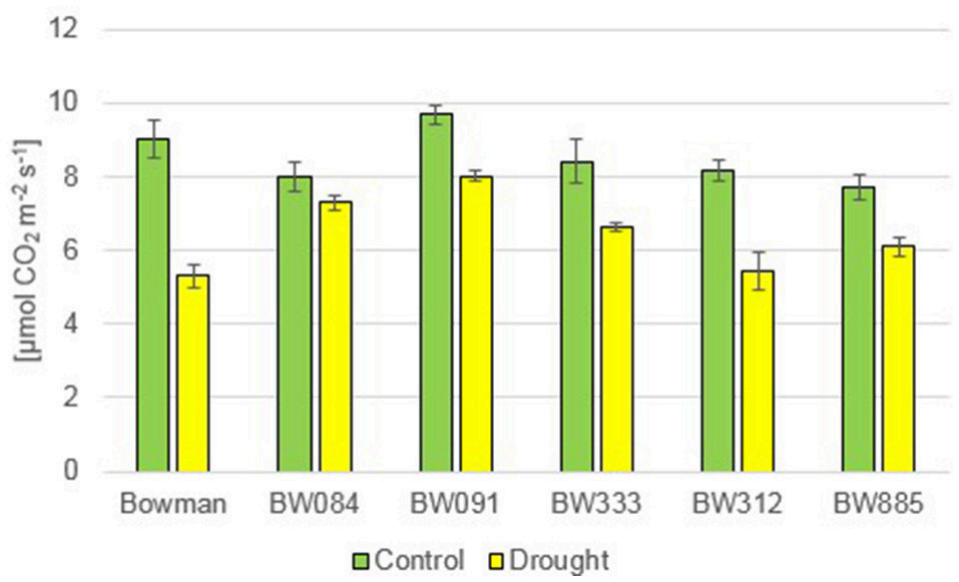
**The photosynthesis rate in the analyzed genotypes under the control and drought conditions**. The mean values of seven replicates of each measurement are presented for each genotype, with error bars representing standard deviation.

In reaction to drought, the semi-dwarf NILs exhibited delayed wilting when compared with the “Bowman” cultivar. Symptoms of leaf wilting were first reported in plants of the “Bowman” cultivar, whereas in the semi-dwarf NILs it was manifested mainly as rolling of leaf blades along the longitudinal axis. The photosynthesis rate (Pn) did not show any significant alteration between the analyzed genotypes under the control condition, and no tendency was observed. As expected, drought caused a decrease in the Pn values in all genotypes. Interestingly, a relative decrease in the Pn value with respect to the control condition was most prominent in the “Bowman” cultivar (decrease by 41.2%) (Figure 11).

**Figure 12:**
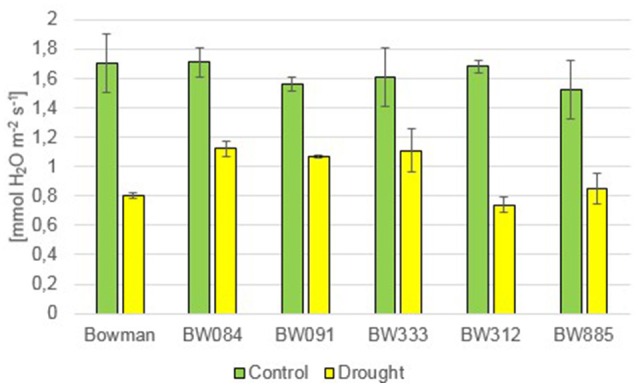
**The transpiration rate in the analyzed genotypes under the control and drought conditions**. The mean values of seven replicates of each measurement are presented for each genotype, with error bars representing standard deviation.

**Figure 13:**
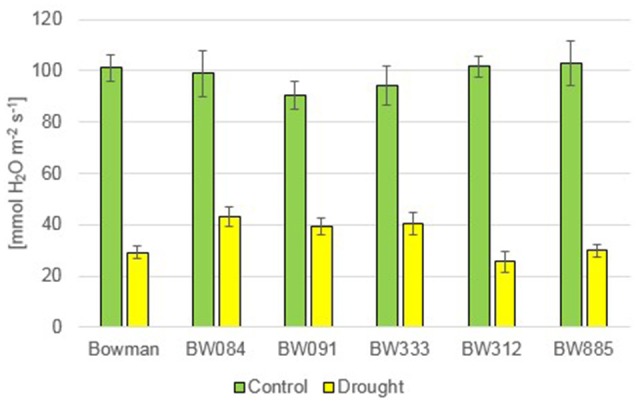
**The stomatal conductance in the analyzed genotypes under the control and drought conditions**. The mean values of seven replicates of each measurement are presented for each genotype, with error bars representing standard deviation.

Under the control condition, no statistically significant difference in the transpiration rate (E) was observed between the genotypes. The drought stress resulted in a decline in the transpiration rate in all genotypes. However, during drought the semi-dwarf NILs were able to maintain transpiration at a similar or higher level when compared with the “Bowman” cultivar (Figure 12). Under the control condition, values of the stomatal conductance (gs) were very similar between the genotypes. As expected, drought caused a sharp decline in the stomatal conductance in all genotypes. However, similarly to the transpiration rate, values of the stomatal conductance in the semi-dwarf NILs were maintained at a similar or even higher level when compared with the “Bowman” cultivar (Figure 13).

## Discussion

In previous studies, mostly exogenous BRs applications were performed, thus the role of the endogenous BRs in the regulation of plant drought response remains controversial and requires further analysis (Jager et al., 2008; Llanes et al., 2016). In the present study the accumulation of two representatives of BRs, CS and 24-epiBL, was found to be significantly stimulated by drought in all analyzed genotypes. This indicated that these two compounds may play a role in the regulation of physiological reaction to the stress condition. It should be kept in mind that CS is suggested as an end product of the BR biosynthesis pathway in monocots, representing the highest biological activity (Kim et al., 2008). Interestingly, in pea (*Pisum sativum*) no substantial change in the CS content was reported under drought stress, however in this species the drought-induced significant increase in the ABA content may be accompanied by a slight increase in the endogenous CS concentration (Jager et al., 2008), which indicates that these effects may be species-dependent. In all analyzed barley genotypes neither BL nor 24-epiCS have been detected in our previous study (Dockter et al., 2014) and in the present assay, what suggests that CS is a major bioactive BR in barley. Noteworthy, the drought-induced accumulation of CS and 24-epiBL is not dependent on functional BR biosynthesis or signaling pathways, as it is shared by all analyzed genotypes. The accumulation of endogenous 24-epiBL under the control condition was found to be specific for the BR-insensitive line BW885. The accumulation of this compound specifically in this NIL was reported in our previous assay (Dockter et al., 2014), however at that time we assumed that it might be an artifact, therefore the data was unpublished. However, in the present research we confirmed that the BR-insensitive line BW885 is capable of accumulating 24-epiBL under the control condition. In the previous research (Dockter et al., 2014) the accumulation of 24-epiBL in the BW885 line was found to be maintained at the higher temperature of plant growth condition (26°C), however it was not stimulated by this temperature stress (data unpublished). In the present study it was shown that the accumulation of 24-epiBL is drought-induced in all analyzed genotypes.

In the study presented here, the accumulation of the C_29_ BR, 28-homoCS, has been detected in relatively large quantities in all analyzed genotypes under control and drought stress conditions. Moreover, a quite interesting phenomenon was observed when the 28-homoCS accumulation was compared between the analyzed genotypes: under the control condition the accumulation of 28-homoCS is inversely correlated with the accumulation of CS in all analyzed genotypes. It was recently reported that in rice the biosynthesis pathway of the C_29_ homoBRs is connected to the C_28_-BR biosynthesis pathway (in which CS is produced) by C-28 demethylation, and the aim of this process is to increase biological activity. Hence, it was concluded that the C_29_-BR biosynthesis is probably an alternative route to the production of endogenous CS in rice (Joo et al., 2015). The C_29_-BR biosynthesis pathway is connected with the C_28_-BR biosynthesis pathway at several steps (Bajguz and Tretyn, 2003; Joo et al., 2015), and it seems to be true also in barley, as the analyzed NILs deficient in the biosynthesis of CS (however, at various steps, because they carry the mutations in the enzymes mediating different reactions during the biosynthesis pathway) accumulate the highest and comparable concentrations of 28-homoCS. Up to now, the occurrence of 28-homoCS was reported only in two monocot crop species: rice (*Oryza sativa*) (Abe et al., 1995) and rye (*Secale cereale*) (Schmidt et al., 1995), and several other species representing various taxonomic groups of the plant kingdom, however our knowledge of the C_29_-BR biosynthesis in plants is still quite limited (Joo et al., 2015). In this paper we show that the accumulation of the C_29_ BR, 28-homoCS, occurs also in barley. The results of our experiments indicate that in contrast to the accumulations of CS and 24-epiBL (representing C_28_ BRs), which were significantly stimulated by drought in all analyzed genotypes, the accumulation of 28-homoCS (representing C_29_ BRs) is not induced by the stress condition. It may be explained by the fact, that biological activity of C_29_ BRs is relatively low in comparison with the biological activity of C_28_ BRs (CS in particular) (Joo et al., 2015), therefore it is likely that the drought-induced physiological reaction of barley plants is mediated by CS, which is the most bioactive form of BRs.

Analysis of the accumulation profile of endogenous GAs performed in the present study indicated that the concentration of GA_4_, one of the major bioactive forms of GAs, under the control condition is lower in all analyzed NILs when compared with the “Bowman” cultivar, however the differences were not found to be statistically significant. BRs are known to regulate biosynthesis of GAs in *A. thaliana*. The Arabidopsis mutants showing abnormalities in the BR signaling and biosynthesis are impaired in the biosynthesis of bioactive GA, which is related with a perturbation in GA biosynthetic gene expression (Unterholzner et al., 2015). BRs regulate expression of GA biosynthetic genes in rice as well (Tong et al., 2014). However, the BR-dependent regulation of GA biosynthesis seems to be species-dependent, since contents of bioactive GAs were not reduced in BR mutants of pea (Jager et al., 2005). Moreover, it should be also kept in mind that the GA biosynthesis is also strongly regulated by environmental factors, including abiotic stresses (Hedden and Thomas, 2012). In our study the drought stress induced a slight increase in the content of GA_4_ in all analyzed genotypes (with respect to the control condition), however differences between the “Bowman” cultivar and the analyzed NILs were not statistically significant. Hence, we conclude that both the BR-deficient and BR-insensitive NILs are able to react to drought with the slight increase in the GA_4_ accumulation, however it does not seem to be a major reaction of barley GA metabolism to the stress condition.

However, homeostasis of another representative of GAs, GA_7_, which is another major bioactive form of this class of phytohormones (Yamaguchi, 2008) seems to be BR-dependent in barley, as under the control condition all analyzed NILs contained significantly lower concentrations of this compound in comparison with the “Bowman” cultivar. Moreover, the endogenous GA_7_ accumulation is also significantly drought-induced in barley, and this stimulation was even more prominent in the analyzed NILs. As a consequence, under the drought condition the endogenous contents of GA_7_ were comparable in all analyzed genotypes. Therefore, our study indicates that in barley GA_7_ is a major representative of GAs, whose concentration is BR-dependent and significantly drought-induced. Hence, we postulate that barley belongs to the same group of plant species as rice (Tong et al., 2014) and Arabidopsis (Unterholzner et al., 2015), in which GA biosynthesis is a BR-regulated process.

Our results indicated that in barley mutations in the analyzed BR-biosynthetic or BR-signaling genes do not affect the endogenous ABA accumulation under the control condition. Similar results were reported in pea (Jager et al., 2008). As expected (Outlaw, 2003; Huang et al., 2008), the drought stress induced a very significant increase in the accumulation of ABA in all analyzed genotypes, therefore we postulate that in barley the perturbations in BR metabolism do not influence the capacity of the mutants to react to the stress with such a significant increase in ABA accumulation. Similar results were obtained in pea (Jager et al., 2008). However, it seems that interdependency between these two phytohormones may be dependent on plant species and on some other factors, like genetic background or gene-specific effects. In tomato (*Solanum lycopersicum*) the ABA concentrations were reduced in a BR biosynthetic mutant (Zhou et al., 2014). In our previous study of two allelic, BR-deficient barley mutants, in which missense substitutions were identified in the *HvDWARF* gene (which has not been taken into account in the present study), ABA concentrations in both mutants were reduced under control and drought condition in comparison with the reference “Delisa” cultivar (Janeczko et al., 2016). We suggest that this result may be specific for the mutations in the *HvDWARF* gene and/or may be an effect of different genetic backgrounds of the two reference cultivars (“Bowman” and “Delisa”). Consequently, it may be a secondary effect of different physiological reactions of these cultivars to the drought stress. Nevertheless, it should be noted that, similarly to the present study, both mutants from the “Delisa” cultivar retained the ability to react to drought stress with a significant increase in the endogenous ABA accumulation (Janeczko et al., 2016). It has been reported in several studies that exogenous BR application increased ABA accumulation, and the effect was more significant under stress conditions (Kurepin et al., 2008; Liu et al., 2009; Yuan et al., 2010; Zhang et al., 2011). However, it should be kept in mind that the drought-induced increase in the accumulation of endogenous ABA may also occur without an exogenous BR treatment (also in this study). Noteworthy, it has been reported in pea that drought causes a significant increase in ABA concentration, however the ability of the plant to increase the ABA accumulation in response to the stress is not affected by BR deficiency, as there was no significant difference in the ABA content between a wild-type genotype and the BR-deficient or BR-insensitive mutants (Jager et al., 2008). Our results in barley are in agreement with this finding. It has been demonstrated that BRs and ABA show an antagonistic relationship in several physiological processes (Zhang et al., 2009), however it is unknown whether there is an antagonistic interaction between these phytohormones in the stress response. It was reported that exogenous BRs may induce the ABA biosynthesis during stress response via an indirect mechanism through a transient H_2_O_2_ production (Zhou et al., 2014), and it added another level of complexity into this inter-hormonal interaction.

It is known that SA is a phytohormone playing a significant role in plant reaction to biotic stress, however recent reports indicate that SA participates also in plant responses to abiotic stresses, including drought (Hayat et al., 2010). However, the exact mechanism of SA involvement in drought response is still not fully understood, because the endogenous SA accumulation is modified by various developmental and physiological factors (Llanes et al., 2016). In the present research we demonstrated that alteration in the SA homeostasis and the significant increase in the endogenous SA accumulation is a physiological reaction of barley to drought stress. Moreover, we postulate that the abnormalities in BR biosynthesis or signaling do not seem to affect the SA homeostasis under the control condition, and do not influence the capability of the barley mutants to react to drought stress with an increase in SA accumulation. The further evidence that endogenous BRs (and the perturbations in their metabolism) do not influence the SA homeostasis was obtained from the measurement of the endogenous concentration of benzoic acid, which is known to be a SA precursor (Chong et al., 2001; Chen et al., 2009). All analyzed genotypes contained a very similar concentration of the endogenous benzoic acid under the control and drought conditions. Moreover, no significant alteration in the benzoic acid content was reported in any of the genotypes in response to the drought stress. Taking into account the significant increase in the endogenous SA accumulation in response to drought, which was reported in this study, we concluded that in barley the drought-stimulated SA accumulation occurs at a step downstream of benzoic acid—the precursor of SA biosynthesis.

It was shown that in barley the regulation of JA homeostasis is a BR-dependent process, as under the control condition all analyzed NILs (both BR-deficient and BR-insensitive) contained significantly lower concentrations of this hormone in comparison with the “Bowman” cultivar. Accordingly, it was previously reported in Arabidopsis and rice that BRs increase the JA content under normal condition (Müssig et al., 2000; Kitanaga et al., 2006). It seems that the BR-JA interplay may be quite complicated, as it was reported that exogenous methylJA application significantly repressed expression of BR biosynthesis genes, and consequently decreased the endogenous BR content (Gan et al., 2015). These results broaden the general view of this inter-hormonal interaction, as it has been previously reported that BRs negatively regulate JA signaling (Kim et al., 2013; Chung et al., 2014). Interestingly, the exogenous methylJA was found to repress the expression of the BR signaling and target genes (Gan et al., 2015). As expected (Huang et al., 2008; Llanes et al., 2016), drought stress induced the significant increase in the endogenous JA content in all analyzed genotypes. Interestingly, both the BR-deficient and BR-insensitive barley mutants retain the capacity of the significant increase in the endogenous JA content in reaction to drought, and relatively to even a higher extent when compared with the “Bowman” cultivar. This increase in the endogenous JA concentration may potentially have a secondary effect, as it was reported in rice that methylJA may stimulate the ABA accumulation (Kim et al., 2009).

Apart from the analysis of homeostasis of various phytohormones, which was described above, some aspects of physiological response of the semi-dwarf NILs to drought were also characterized. In reaction to drought, the semi-dwarf mutants exhibited delayed wilting when compared with the “Bowman” cultivar. Accordingly, in the recent report in which two semi-dwarf BR-deficient mutants along with the reference “Delisa” cultivar were exposed to drought, it was noted that the stress condition negatively affects growth of the tall “Delisa” cultivar to much greater extent than that of semi-dwarf mutants (Janeczko et al., 2016). These observations are quite intriguing, as they seem to be congruent with the recent report indicating that semi-dwarf rice mutant with erect leaves may show prolonged tolerance to drought (Ferrero-Serrano and Assmann, 2016). Moreover, it has been reported that reduction of BR responses improves plant tolerance to drought (Northey et al., 2016). In the present study, the photosynthesis rate (Pn) did not show any significant alteration between the analyzed genotypes under the control condition. Interestingly, the drought-induced relative decrease in the Pn value with respect to the control condition was most prominent in the “Bowman” cultivar. These results seem to be congruent with an analysis of net photosynthesis in the semi-dwarf rice mutant *d1*. Under drought condition, wild-type plants exhibited a steeper decrease in CO_2_ fixation when compared with the *d1* mutant (Ferrero-Serrano and Assmann, 2016). Abnormalities in BR metabolism do not seem to negatively affect transpiration rate and stomatal conductance in the semi-dwarf NILs under the control condition. Similar results were obtained for other BR-related barley and rice mutants (Ferrero-Serrano and Assmann, 2016; Janeczko et al., 2016). In response to drought, the semi-dwarf NILs maintained the transpiration rate and stomatal conductance at a similar or even higher level when compared with the “Bowman” cultivar. As stated above, the abnormalities in BR metabolism result in phenotypic alterations only in the above-ground part of the analyzed NILs, and consequently in the semi-dwarf stature, however having no effect on root architecture (Dockter et al., 2014). It may be of significant importance for water use efficiency, as root-to-shoot biomass ratio is an important factor influencing drought tolerance (Ferrero-Serrano and Assmann, 2016).

The present study provided a comprehensive analysis of the role of endogenous BRs (and the abnormalities in their metabolism) in regulation of the homeostasis of other phytohormones, and plant response to drought stress. However, there are still many questions, which should be answered and require a further study. Therefore, we have already commenced a further research, which is aimed at a multi-directional physiological analysis of these genotypes in reaction to drought. Another objective of this research is also a verification whether these semi-dwarf barley mutants may constitute an alternative for future breeding programs aimed at development of cultivars with an improved drought tolerance, and adapted to the approaching climate changes.

## Author contributions

DG: Conceived the project; DG, AJ, and IS: Planned the research; DG, AJ, MD, EP, and JO: Collected and analyzed the data; DG: Interpreted the data and wrote the manuscript. All the authors gave the final approval for submission of the manuscript.

## Funding

This work was supported by funds from the Polish Ministry of Agriculture and Rural Development (grant no. HOR hn-801/11/14-PO-0114-002), and partly from the Ministry of Education, Youth and Sports of the Czech Republic (grant no. NPUI nr. LO1204).

### Conflict of interest statement

The authors declare that the research was conducted in the absence of any commercial or financial relationships that could be construed as a potential conflict of interest.
